# Maternal postpartum health and its impact on health and development of young children

**DOI:** 10.4069/whn.2024.03.30

**Published:** 2024-05-24

**Authors:** Lorraine O. Walker

**Affiliations:** School of Nursing, The University of Texas at Austin, Austin, TX, USA

## Introduction

With the increasing global prevalence of non-communicable diseases, such as diabetes [1], maternal health after childbirth rarely garners comparable attention, except for occurrence of maternal mortality. Indeed, it is devasting to hear that in 2020, it is estimated that 287,000 women globally lost their lives from pregnancy-related causes that occurred either during pregnancy, childbirth, or the first 42 days after giving birth [2]. While most of those deaths occurred in middle- and low-resource countries, at least one high-resource country—the United States—has actually experienced a rise in maternal mortality in recent years [3]. That rise in the United States has spurred new research initiatives, and a wide range of clinical and health system changes and community partnerships focused on reducing maternal mortality.

Though most women survive pregnancy, childbirth, and the postpartum period, they may still face challenges to their health that often receive little attention. These conditions may take many forms, ranging from issues related to mental health and well-being, such as postpartum depression, to challenges in physical health and functioning, such as urogenital conditions [4,5]. Such mental and physical conditions reduce the quality of life for women and may continue after the first 6 postpartum weeks [4,5], when pregnancy-related healthcare has ceased. That women’s health after the early postpartum weeks has received limited attention, except perhaps for postpartum depression, is consistent with the overall disparate attention to women’s health problems compared to those of men [6].

As debilitating and stressful as certain postpartum mental and physical health conditions may be for women themselves, it is important to also consider how such maternal health problems may affect the health and development of their children. The division of healthcare and scientific literatures dealing with maternal and child health into obstetrics and pediatrics may partly contribute to potential gaps in our understanding of the effects that maternal health may have on infants and children. Thus, the aim of this paper is to examine briefly scientific literature on the relationship of postpartum maternal health to the health and development of infants and young children in order to identify issues, gaps, and opportunities.

## Postpartum mental health and its relationship to child health and development

Among postpartum mental health conditions, postpartum (also called postnatal) depression stands out both in terms of its global prevalence of 17% [7] and the large volume of scientific attention it has received over time [8,9]. What is clear from reviews looking comprehensively at postpartum depression is that it is a complex phenomenon that is best understood from an integrative perspective that incorporates biological and psychosocial contributing factors [8]. Symptoms of postpartum depression may include sadness, anhedonia, sleep problems, difficulty concentrating, guilt, and anxiety that arise after pregnancy that are not a continuation of pre-existing depression [9]. The gold standard for diagnosing postpartum depression is a clinical interview by a healthcare provider with training in mental health. However, a number of studies also have utilized self-report depressive symptoms scales that have cutoff scores for risk of depression, such as the Edinburgh Postnatal Depression Scale (EPDS) [8,9].

Although a devasting condition for women to experience, both postpartum depression and elevated depression symptoms measured by scales are associated with infant and child adverse outcomes [9]. This is especially true if maternal depression is untreated. In a comprehensive review of 67 studies of postpartum depression, Slomian et al. [9] reported the maternal postpartum depression was associated with adverse effects on infant growth, physical health, and multiple domains of development. The review showed that effects of depression on infant growth were generally more marked among infants in lower-resource countries, with infant weight lower when mothers were depressed compared to when mothers were not. Two child health indicators, diarrhea and infant mortality were higher among children of depressed mothers, primarily in studies of samples in lower-resource countries.

In regard to the effects of postpartum depression in a higher-resource country, a recent population-based study in Israel provides insight into the impact of maternal depression on children’s development during the first 2 years of life [10]. In this sample of over 90,000 postpartum women, 4.7% were screened positive for depression on the EPDS at 6 to 9 weeks after giving birth. Infants’ achievement of developmental milestones in four domains (language, personal social, fine motor and gross motor) were monitored at periodic clinic visits from 1 to 24 months of age. Over the 2 years of monitoring, postpartum depression was associated with less achievement of developmental milestones in all four domains, but most strongly with developmental delays in language and personal social development [10].

A study in the United Kingdom that drew on data from the Avon Longitudinal Study of Parents and Children sheds light on the behavioral development of young children with depressed mothers [11]. Postpartum depression was categorized as moderate, marked, and severe based on EPDS scores of 9,848 mothers. Behavioral problems were assessed by maternal reports of children’s behaviors at 3.5 years on the Rutter Total Problems Scale. Regardless of the level of depression at 2 months postpartum, it was associated with approximately twice the odds of children having behavioral problems at age 3.5 years. When depression was severe and persisted after the immediate postpartum period, it had more adverse effects on children’s behavioral problems [11].

While the literature on postpartum depression and child outcomes is both vast and complex, it is clear that such depression has the potential to affect physical health, development, and psychosocial adjustment in young children. The extent of that impact may vary with some effects, such as effects on child physical health, being most adverse among those in lower-resource countries. Clearly the larger caregiving milieu and resources in the family can lessen or magnify effects of maternal depression on young children. However, early childhood is a vulnerable period of development during which maternal depression may put child survival at risk in low-resource countries. Even in more resource-rich countries, postpartum depression may put young children’s well-being at risk across multiple domains.

## Postpartum physical health and its relationship to child health and development

Maternal mortality is the most dire health outcome for women related to childbirth and it additionally may have a major impact on infants, children, and their families. That impact is most severe in low-resource countries where services are limited. For example, a longitudinal study examined child survival in a rural region of Ethiopia [12]. When mothers died (from maternal and non-maternal causes) shortly after birth or during the first year after childbirth, their infants were more likely also to die compared to infants whose mothers survived. And those infant deaths were more likely to occur in the first month after birth [12]. Deaths of young infants were often associated with being undernourished, an outcome of loss of maternal care and breastfeeding [13].

The adverse impact of maternal deaths on infant survival replicates findings of earlier global studies [12]. Surviving children of mothers who died during the first year after childbirth also were less likely to attend school than children with surviving mothers, with girl children often taking on household responsibilities in the family [12,13]. These findings exemplify the importance of maternal survival to child survival and developmental opportunities in low-resource countries. And those negative effects may extend beyond the health and survival of children. For example, a study in Ghana showed the overall effects of maternal death on the family were widespread and associated with financial hardship, grief among survivors, and often placement of surviving children with other family members [14].

More generally, what is known about the impact of maternal general and physical health on child health and development? The study of this topic primarily has focused on adolescents rather than younger children [15]. But several studies highlight the vulnerability of young children. An early study in the United States using the 1988 Maternal and Infant Health Survey, a longitudinal population-based dataset, examined multiple indicators of maternal health and outcomes of children. Mothers’ physical health after pregnancy, assessed by number of doctor visits or hospitalizations, was significantly associated with poorer child health ratings at age 3 years [16].

Two more recent Australian studies provide additional insights into the relationship of maternal general and physical health on young children’s health and development. The first study used data from 5,019 infants born in 2002 to 2003 in the Longitudinal Survey of Australian Children. Results showed that mothers’ less positive ratings of their health were associated with less positive ratings of their infants’ health [17].

The second study utilized linked administrative data on mothers’ and fathers’ health (based on having a chronic disease) with the 2009 Australian Early Development Census. Associations assessed were between parental health and the developmental outcomes at 5.5 years of 19,071 children [15]. During the study period, prevalence of a chronic disease was 7.3% for mothers and 6.8% for fathers. Having a mother with a chronic disease increased the odds of a daughter being classified as developmentally “vulnerable/at risk” in three domains: physical health and well-being, social competence, and communication skills and general knowledge. Having a mother with a chronic disease increased the odds of a son being classified as developmentally “vulnerable/at risk” only in the language and cognitive skills domain. However, having a father with a chronic illness was not associated with either daughters or sons being classified as “vulnerable/at risk” on any developmental domain [15]. These findings underscore the importance of maternal health to child health, especially for girl children.

## Discussion

Overall, the studies cited here give snapshots of the varied impacts that maternal health problems—both mental and physical—may have on health and development of young children. My intent in this paper was not to do a systematic review of this topic, but to highlight key findings and draw attention to the potential effect that maternal mortality and poor health may have on children. An extensive review of maternal mortality and morbidity globally is available elsewhere, which also considers human immunodeficiency virus and its effects [18].

While maternal mortality and its effects on young children are profound in lower-resource countries, in higher-resource countries maternal chronic diseases may pose the more common risk to children’s health and development. However, a common thread in both situations is the need for support and services from the healthcare sector and the larger community. The support and services needed to protect children from poor development and health outcomes will vary widely depending upon family and caregiver needs to sustain a nurturing, safe, and healthy environment for young children. For example, in the study cited earlier in Ghana of family survivors after a maternal mortality, family and community support was high through the funeral but dissipated afterwards [14]. Thus, systems of care in the healthcare and social care sectors that can fill gaps are needed, whether in primary care or integrative maternal and child health care. And those care services would be most effective if provided with a family focus to provide a robust scaffolding for child health and development.

Nurses and midwives can and do play an important role in protecting maternal health during pregnancy, birth, and the postpartum period through their direct care, counseling, and linking women and young children to community services. Such nursing care is a critical foundation for supporting child health because of pivotal role of maternal health to child health, as briefly shown here. Because the current resources of families, communities, and countries may vary, interventions and programs that nurses and midwives may develop, or advocate for, will need to be tailored to the situational context. Such services may be especially important for girl children [15].

In conclusion, maternal mortality and mental and physical health have important consequences for health and development of infants and young children. Maternal mortality poses more extensive threats to maternal health and consequently child health and development in lower-resource countries compared to higher-resource ones. However, mental health conditions have no economic boundaries and affect women in varying contexts thereby affecting child health and development. More attention in practice and research needs to be given to how to protect maternal health in order to protect the health and development of young children.
